# Why do authors derive new cardiovascular clinical prediction rules in the presence of existing rules? A mixed methods study

**DOI:** 10.1371/journal.pone.0179102

**Published:** 2017-06-07

**Authors:** Jong-Wook Ban, Emma Wallace, Richard Stevens, Rafael Perera

**Affiliations:** 1 Evidence-Based Health Care Programme, Centre for Evidence-Based Medicine, University of Oxford, Oxford, United Kingdom; 2 HRB Centre for Primary Care Research, Royal College of Surgeons in Ireland, Dublin, Ireland; 3 Nuffield Department of Primary Care Health Sciences, Medical Science Division, University of Oxford, Oxford, United Kingdom; York University, UNITED KINGDOM

## Abstract

**Background:**

Researchers should examine existing evidence to determine the need for a new study. It is unknown whether developers evaluate existing evidence to justify new cardiovascular clinical prediction rules (CPRs).

**Objective:**

We aimed to assess whether authors of cardiovascular CPRs cited existing CPRs, why some authors did not cite existing CPRs, and why they thought existing CPRs were insufficient.

**Method:**

Derivation studies of cardiovascular CPRs from the International Register of Clinical Prediction Rules for Primary Care were evaluated. We reviewed the introduction sections to determine whether existing CPRs were cited. Using thematic content analysis, the stated reasons for determining existing cardiovascular CPRs insufficient were explored. Study authors were surveyed via e-mail and post. We asked whether they were aware of any existing cardiovascular CPRs at the time of derivation, how they searched for existing CPRs, and whether they thought it was important to cite existing CPRs.

**Results:**

Of 85 derivation studies included, 48 (56.5%) cited existing CPRs, 33 (38.8%) did not cite any CPR, and four (4.7%) declared there was none to cite. Content analysis identified five categories of existing CPRs insufficiency related to: (1) derivation (5 studies; 11.4% of 44), (2) construct (31 studies; 70.5%), (3) performance (10 studies; 22.7%), (4) transferability (13 studies; 29.5%), and (5) evidence (8 studies; 18.2%). Authors of 54 derivation studies (71.1% of 76 authors contacted) responded to the survey. Twenty-five authors (46.3%) reported they were aware of existing CPR at the time of derivation. Twenty-nine authors (53.7%) declared they conducted a systematic search to identify existing CPRs. Most authors (90.7%) indicated citing existing CPRs was important.

**Conclusion:**

Cardiovascular CPRs are often developed without citing existing CPRs although most authors agree it is important. Common justifications for new CPRs concerned construct, including choice of predictor variables or relevance of outcomes. Developers should clearly justify why new CPRs are needed with reference to existing CPRs to avoid unnecessary duplication.

## Introduction

Healthcare professionals regularly face diagnostic and prognostic uncertainties. Clinical prediction rules (CPRs) can help address these uncertainties and help make evidence-based decisions using information from individual patients [1–4]. The following steps are needed to develop a CPR [2, 4, 5]. Firstly, a CPR is constructed in a derivation study using variables predictive of an outcome. Secondly, the generalizability of the CPR should be tested in external validation studies. Lastly, the ability of the CPR to improve clinical outcomes and efficiency should be examined in impact analyses.

Many CPRs have been developed for various cardiovascular problems [6–8]. These cardiovascular CPRs are better recognized and more commonly used by general practitioners (GPs) compared with CPRs in other clinical areas [9]. However, the development of cardiovascular CPRs has been disproportionately focused on some clinical domains while many have not been externally validated and few have been assessed in impact studies [6, 7, 10]. For example, a systematic search identified 64 similar prognostic CPRs for congestive heart failure and 50 modified CPRs that consisted of many overlapping predictors [11]. At the same time, many developed CPRs are not utilized. A survey of general practitioners from the UK and a systematic review of international clinical guidelines from selected clinical domains in which CPRs were known to have been published demonstrated that in most clinical domains, CPRs were seldom adopted by guidelines or used in practice [9].

Before new research is undertaken, what is known about the subject should be carefully reviewed to identify pertinent research questions, choose optimal study designs and avoid unnecessary duplication [12–14]. Researchers developing new CPRs should also clearly establish the need by examining all existing CPRs for the clinical problem. Producing CPRs by analyzing existing data without assessing what is already available may lead to many redundant CPRs. The Transparent Reporting of a multivariable prediction model for individual Prognosis Or Diagnosis (TRIPOD) statement published in 2015 recommends authors present a rationale for developing a new CPR with references to existing CPRs [15]. However, it is not yet known whether developers of cardiovascular CPRs justify need by reviewing existing CPRs.

We examined firstly whether authors cited existing cardiovascular CPRs in derivation studies. To understand why authors proceed to develop a new CPR when previous CPRs exist, we then studied the stated insufficiencies of existing cardiovascular CPRs, according to the authors of derivation studies. Lastly, a survey was conducted to examine why some authors cited existing cardiovascular CPRs and others did not.

## Materials and methods

### Information source and selection of derivation studies

We used cardiovascular CPRs included in the International Register of Clinical Prediction Rules for Primary Care. The development of the international register and its contents have been reported previously in detail [7]. The international register is composed of 745 articles retrieved by searching Medline (PubMed) for CPR literature published between 1980 and 2009, searching secondary sources, and contacting experts in CPR research [7]. It contains 434 CPRs across 17 clinical domains according to the International Classification of Primary Care, Second Edition (ICPC-2) [7, 16]. The cardiovascular domain includes 138 CPRs for various cardiovascular problems in 17 clinical sub-domains such as acute myocardial infarction (K75), heart failure (K77), and pulmonary embolism (K93).

All articles included in the cardiovascular domain of the international register were considered. Eligibility was not restricted to any derivation year, population, setting or language used. For an article to be eligible, authors must have developed a new cardiovascular CPR from the ground up by selecting a unique set of predictor variables. Articles were excluded if they exclusively updated existing cardiovascular CPRs: (1) the intercept and coefficients of an existing CPR were modified using the calibration intercept and slope (recalibration), (2) the intercept and coefficients of an existing CPR were recalculated (re-estimation), or (3) some predictors of an existing CPR were added, removed, or replaced (modification) [17–21]. We also excluded review articles that presented CPRs without describing how they were developed. Articles that lacked an introduction section were also excluded (e.g. conference abstracts).

### Descriptive review of derivation studies

Two authors (JB and EW) independently extracted data. Disagreements were adjudicated by discussion, and if agreement could not be reached by a third reviewer. For each eligible derivation study, the following information was recorded: (1) bibliographic data (authors and publication year), (2) the country where the CPR was developed, (3) the type of CPR (diagnostic or prognostic), (4) the sub-domain of the international register that the CPR belongs to, and (5) the citation of existing cardiovascular CPR.

A CPR was classified as a diagnostic rule if it assessed the presence of a condition at the time of the prediction [15, 22]. A CPR was classified as a prognostic rule if it assessed a future occurrence of an outcome after the prediction [15, 22]. We considered a derivation study to be citing an existing CPR when an introduction section included a sentence discussing an existing cardiovascular CPR and a relevant bibliographic reference. The derivation studies were divided into 3 groups according to the citation of existing cardiovascular CPR: derivation studies that (1) cited existing CPR, (2) did not cite any existing CPR, and (3) declared there was no CPR to cite for the cardiovascular condition of interest.

### Content analysis

We examined the introduction sections of the derivation studies that cited existing cardiovascular CPRs. A thematic content analysis was conducted to produce a typology of the stated insufficiencies of existing CPRs according to the authors of these derivation studies [23]. The thematic content analysis focused on manifest content rather than latent content because reliably interpreting implicit meaning of content could be difficult. Since there was no prior knowledge about why new cardiovascular CPRs are created, we used an inductive approach to develop a coding scheme by allowing themes to emerge from the examination of data.

Derivation studies that cited existing cardiovascular CPRs were imported to Nvivo for Mac (QSR International Pty Ltd). To become familiarized with data, one of the authors (JB) repeatedly read the introduction section of each derivation study. Then, the author (JB) identified sentences that contained any reason why an existing CPR was insufficient to address the cardiovascular problem. A preliminary code was created using key words from each sentence.

After creating preliminary codes for 30% of randomly sampled derivation studies, a coding scheme was generated by combining preliminary codes with an overlapping theme. The remaining derivation studies were coded according to the coding scheme. A new code was generated when there was content that described an insufficiency of existing cardiovascular CPRs but was not compatible with any existing codes. Previously coded derivation studies were re-coded using the updated coding scheme. Data within each code were reviewed to confirm that they were consistent with the assigned code. The code was divided when an inconsistency was found. Similar codes were organized into categories.

The results of the content analysis are presented by describing each category with associated texts from derivation studies. The number of derivation studies that provided each category of reasons as to why existing cardiovascular CPRs were insufficient is reported.

### Survey of authors

A survey was conducted to understand why some authors cited existing cardiovascular CPRs in their derivation studies and others did not. The Medical Sciences Interdivisional Research Ethics Committee (MS IDREC) of the University of Oxford conducted an ethical review and approved the survey (reference number: MS-IDREC-C1-2015-161).

Target survey participants were the corresponding authors of the derivation studies in the descriptive review described above. When a corresponding author could not be reached, we attempted to contact the first author. E-mail and postal addresses of the potential target participants were recorded from each derivation study and were updated by searching in PubMed and the internet.

In November 2015, each target participant was contacted first by sending a personalized e-mail which included a link to an online questionnaire and a participant invitation letter. A second e-mail was sent if the author did not respond within eight weeks. We posted a personalized survey packet to authors whose e-mail address was not known or who did not respond to a third e-mail invitation. Authors with known postal addresses in the United States (US), Canada or United Kingdom (UK) were contacted. The survey packet included a participant invitation letter, a questionnaire, and a stamped return envelope. The participant invitation letter outlined the purpose of the survey, why the author was chosen, the length of survey, implied consent by completing, confidentiality of data collected, and how to raise any concern.

The survey was developed by considering possible reasons why authors did not discuss existing cardiovascular CPRs. These include: (1) authors were not aware of any existing cardiovascular CPR, (2) authors did not search for existing cardiovascular CPRs, and (3) authors did not believe citing exiting cardiovascular CPR was important. The final survey consisted of three closed-ended questions (S1 Appendix). It was piloted among researchers from the Nuffield Department of Primary Care Health Sciences, University of Oxford, who had experience in developing CPRs.

Participants were asked to confirm they had read and understood the information about the survey by checking a box. They were also required to enter a unique alphanumeric code before starting the survey. No incentive was offered for participating.

### Statistical analysis

The characteristics of derivation studies were reported by absolute and relative frequencies for categorical variables. For non-normally distributed continuous variables, the characteristics of derivation studies were presented with medians and interquartile ranges. The answers to survey questions were presented with absolute and relative frequencies. We used Fisher’s exact test to assess the null hypothesis that answers to survey questions are same between derivation studies that cited existing CPR, did not cite any existing CPR, and declared there was no CPR to cite. Stata (*Release 14*. College Station, TX: StataCorp LP) was used for all analyses.

## Results

### Review of derivation studies

The cardiovascular domain of the international register of CPRs contained 138 CPRs from 131 articles. Of the 131 articles, 46 articles were excluded: 35 articles updated existing CPRs, 10 that presented CPRs in reviews or guidelines without describing how they were developed, and one conference abstract without an introduction section. Eighty-five derivation studies met the inclusion criteria. Of these, 48 derivation studies (56.5%) cited at least one existing CPR, 33 derivation studies (38.8%) did not cite any existing CPR, and 4 derivation studies (4.7%) declared there was no existing CPR to cite. Characteristics of the derivation studies are presented in Table 1.

**Table 1:** Characteristics of derivation studies according to the citation of existing cardiovascular prediction rule.

Characteristics	Cited existing CPR, n = 48 (%)	Did not cite existing CPR, n = 33 (%)	No existing CPR to cite, n = 4 (%)
**Publication year**			
Before 1980	1 (2.1)	0 (0.0)	0 (0.0)
1980–1989	6 (12.5)	6 (18.2)	1 (25.0)
1990–1999	11 (22.9)	11 (33.3)	0 (0.0)
2000–2009	30 (62.5)	16 (48.5)	3 (75.0)
**Location**			
USA	20 (41.7)	15 (45.5)	3 (75.0)
UK	7 (14.6)	5 (15.2)	0 (0.0)
Europe	6 (12.5)	5 (15.2)	1 (25.0)
Canada	4 (8.3)	1 (3.0)	0 (0.0)
Multiple	7 (14.6)	5 (15.2)	0 (0.0)
Other	4 (8.3)	2 (6.1)	0 (0.0)
**Type of CPR**			
Diagnostic	22 (45.8)	19 (57.6)	1 (25.0)
Prognostic	26 (54.2)	14 (42.4)	3 (75.0)
**Sub-domain**			
K90 Stroke/cerebrovascular accident	8 (16.7)	5 (15.2)	1 (25.0)
K22 Risk factor cardiovascular disease	8 (16.7)	3 (9.1)	0 (0.0)
K77 Heart failure	2 (4.2)	7 (21.2)	0 (0.0)
K93 Pulmonary embolism	6 (12.5)	2 (6.1)	1 (25.0)
K74 Ischemic heart disease with angina	6 (12.5)	1 (3.0)	0 (0.0)
K75 Acute myocardial infarction	6 (12.5)	1 (3.0)	0 (0.0)
K94 Phlebitis/thrombophlebitis	4 (8.3)	2 (6.1)	0 (0.0)
K01 Heart pain	2 (4.2)	3 (9.1)	0 (0.0)
K92 Atherosclerosis/PVD	2 (4.2)	2 (6.1)	1 (25.0)
K76 Ischemic heart disease without angina	3 (6.3)	1 (3.0)	0 (0.0)
K99 Cardiovascular disease other	0 (0.0)	2 (6.1)	0 (0.0)
K70 Infection of circulatory system	0 (0.0)	1 (3.0)	0 (0.0)
K78 Atrial fibrillation	0 (0.0)	0 (0.0)	1 (25.0)
K80 Cardiac arrhythmia NOS	0 (0.0)	1 (3.0)	0 (0.0)
K84 Heart disease other	0 (0.0)	1 (3.0)	0 (0.0)
K86 Hypertension uncomplicated	0 (0.0)	1 (3.0)	0 (0.0)
K89 Transient cerebral ischemia	1 (2.1)	0 (0.0)	0 (0.0)

CPR, clinical prediction rule; PVD, peripheral vascular disease.

Of 85 derivation studies of cardiovascular CPRs, 12 reported more than one CPR with different number of predictor variables. The median number of predictor variables included in cardiovascular CPRs was 6 (IQR of 4 to 8 when counting the smallest final models and 4.5 to 9 when counting the largest final models). The median publication year for 48 studies that cited existing CPR was 2002 (IQR 1995, 2005) and for 33 studies that did not cite existing CPR was 1999 (IQR 1995, 2002). There was only one derivation study published before 1980 [24]. The majority of derivation studies (57.6%) were published after 2000. The proportion of derivation studies that cited existing cardiovascular CPRs increased from 46.2% between 1980 and 1989, to 50.0% between 1990 and 1999, and to 61.2% between 2000 and 2009.

Fifty derivation studies (58.8%) were conducted in the US or the UK. These studies included 218,447 participants (87.1% of all participants). The median numbers of participants in the derivation studies were 1,253 (IQR 526, 5711). The largest derivation study was conducted in the UK and included 1,897,727 participants [25]. The smallest derivation study was from India and included 43 participants [26].

### Content analysis

A coding scheme was created by reviewing the introduction sections of 13 randomly selected derivation studies that cited existing CPRs. This initial coding scheme included 21 codes each representing a unique stated insufficiency of existing CPRs (e.g. important predictors were missed, predictors were dichotomized, predictors were not available routinely). While coding 31 remaining derivation studies, four more codes were identified and the coding scheme was updated. Using the updated coding scheme, all 44 derivation studies were re-coded.

The following five categories of insufficiency of existing CPRs emerged when linked codes were aggregated and organized in a hierarchical structure: (1) derivation related, (2) construct related, (3) performance related, (4) transferability related, and (5) evidence related insufficiencies. In addition, some studies simply mentioned existing cardiovascular CPRs without discussing why they were insufficient. The categories of insufficiencies reported in derivation studies of cardiovascular CPRs are presented in S1 Data.

#### (1) Derivation related insufficiency

Five studies (11.4%) stated that existing cardiovascular CPRs had insufficiencies related to their derivation process [24, 27–30]. Four studies stated that the derivations were conducted using biased methods (e.g. selection bias) [24, 28–30] and one study stated that the existing CPRs were developed without the intention of clinical use [27]. For example, Wang et al. [29] reported that the method used to derive one of the prognostic rules for atrial fibrillation (AF) was flawed:

“They tested this scheme using Medicare claims data from patients who were hospitalized for AF but did not receive anticoagulation therapy. A potential limitation of this approach is selection bias, because clinical features associated with nonuse of warfarin or hospitalization for AF are likely to influence stroke risk. Also, some strokes may be missed by hospital discharge data if they are small, immediately lethal, or improperly coded.”

#### (2) Construct related insufficiency

Thirty-one derivation studies (70.4%) stated that existing CPRs had insufficiencies related to their construct [25, 28–57]. Twenty derivation studies (45.4%) mentioned problems with predictor variables of existing CPRs including predictor variables were missed, unavailable in daily clinical practice, clinically insensible, inaccurate, irreproducible, not standardized, not clearly defined, or dichotomized [25, 28, 30–32, 35, 36, 39, 41, 43–46, 48–51, 54, 55, 57]. The following quotations illustrate some of the issues related to predictor variables:

“[T]he Framingham algorithm does not include factors such as social deprivation, body mass index, family history of cardiovascular disease, and current treatment with antihypertensives.”

[25]

“A more fundamental limitation is that the MMPS, and other currently available analogous mortality predictors, use clinical information from the first 24 hours or more not available upon presentation to the hospital,… they cannot be used immediately in the real-time clinical setting.”

[35]

Twelve derivation studies argued that the existing CPRs’ outcomes were irrelevant [29, 33, 34, 39, 40, 42, 47, 48, 50, 52, 53, 56]. One example is shown here:

“Available risk scores do not include mortality as an end point although studies have indicated that AF is an independent risk factor for death as well as stroke and therapies for AF may affect mortality.”

[29]

There were five derivation studies that described issues with presentation of CPRs (e.g. does not produce absolute risk) [25, 29, 42, 49, 51]. Eight studies discussed difficulties of using existing CPRs due to complex calculations, need for a calculator or computer, and large numbers of predictor variables [30, 31, 33, 35, 37, 38, 40, 48].

#### (3) Performance related insufficiency

Authors of 10 derivation studies (22.7%) stated that existing CPRs performed disappointingly [25, 28, 30, 35, 37, 53, 56, 58–60]. Three of these studies discussed inadequate performance of existing CPRs in a subgroup of population (e.g. socially deprived) [25, 35, 60]. Subramaniam et al. [30] justified developing the Hamilton score by highlighting the inaccuracy of Wells score for Deep Vein Thrombosis (DVT):

“The modified Wells score has limitations in discriminating patients likely to have DVT and those unlikely to have DVT. In a study involving 1,096 ambulatory outpatients,… identified… 495 patients (45.2%) as likely to have DVT using the modified Wells score. This is despite the ambulatory population, which is expected to have a lower risk for DVT than hospital inpatients.”

#### (4) Transferability related insufficiency

Authors of 13 derivation studies (29.5%) stated that existing CPRs had limitations in their transferability [25, 28, 29, 36, 43, 47, 50, 56, 61–65]. Seven derivation studies discussed transferability constraints due to differences in key characteristics of participants such as age, sex, and co-morbidities [25, 29, 36, 43, 61, 63, 65]. Five studies reported that differences in disease spectrum may hinder CPR transferability [29, 36, 50, 56, 65]. Some studies discussed dissimilarities in disease prevalence [25, 47, 61] and setting [28, 62, 64] as potential obstacles. Authors of one derivation study discussed concern about using an existing CPR developed in a different setting:

“These studies have been made of selected groups of patients in rehabilitation units. The results therefore may not be applicable to general hospital stroke admissions.”

[62]

#### (5) Evidence related insufficiency

Eight derivation studies (18.2%) cited paucity of evidence in the development of existing CPRs as the reason to create a new rule [25, 27, 31, 37, 47, 53, 57, 61]. Lack of validation and difficulties in updating the CPR in a local population were frequently cited [27, 37, 47, 57, 61]. Uncertainties regarding the impact [25, 53] and lack of uptake in clinical practice [27, 31] were also mentioned.

Lack of validation of existing CPRs was the reason for deriving a new CPR in the following example:

“Although pregnancy is recognized as a risk factor for venous thrombosis, no prospective studies validate the use of current diagnostic strategies for DVT.”

[61]

#### (6) Simple citation

Authors of three derivation studies (6.8%) simply cited existing CPRs without clearly explaining why these CPRs were insufficient and a new CPR was needed [26, 66, 67]. For example, authors of the derivation study in the following example just listed available prediction models without stating why they needed to develop a new CPR:

“Two stroke risk prediction models have been published: the Framingham Study model, and the Israeli Ischemic Heart Disease Project (IIHD) model.”

[67]

### Survey of authors

**Fig 1:**
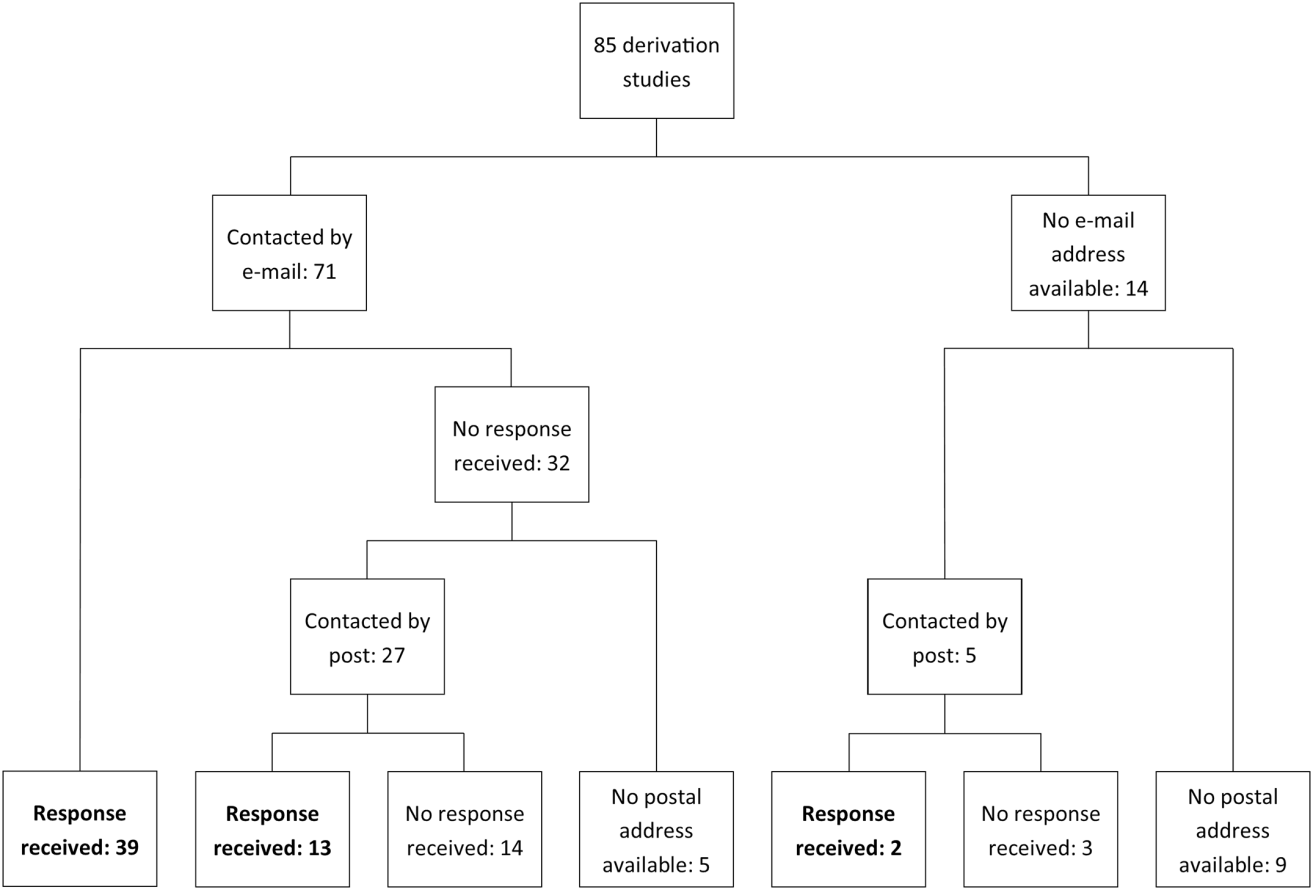
Flow diagram.

Of the 85 derivation studies in the review, we were able to contact the authors of 76 derivation studies by e-mail, post or both (Fig 1). Authors of 54 derivation studies responded (39 by e-mail and 15 by post) giving a response rate of 71.1%. Characteristics of derivation studies where a response from an author was received, authors were contacted but no response was received, and no author could be contacted are presented in S1 Table.

The results of the author survey are presented in Table 2. Of 54 authors who responded, 25 (46.3%) indicated they were aware of existing CPRs that addressed the same clinical problem at the time of derivation. We found that 33.3% of authors who did not cite existing CPR at the time of derivation stated that they were aware of existing CPR at the time of survey. There was no clear association between stated awareness of existing CPRs and citation of these rules (Fisher’s exact *p* = 0.11). To become aware of existing CPRs, 29 authors (53.7%) conducted either a full or a limited systematic review and 15 authors (27.8%) only used non-systematic methods (searching the team’s reference collection or consulting experts). Nine authors (16.7%) did not search for existing CPRs. Type of search method was not clearly associated with citation of existing CPRs (Fisher’s exact *p* = 0.19). Most authors (90.7%) thought citing existing CPRs in a derivation study was either very important or somewhat important. There was no clear evidence that authors who indicated it was important to cite existing CPRs were more likely to do so (Fisher’s exact *p* = 0.71).

**Table 2:** Survey responses according to the citation of existing cardiovascular prediction rule.

Question	Cited existing CPR, n = 34 ^a^ (%)	Did not cite existing CPR, n = 18 (%)	No existing CPR to cite, n = 2 (%)
***1***. ***At the time of derivation***, ***were you aware of any existing prediction rules that addressed the same problem*?**			
Yes	19 (57.6)	6 (33.3)	0 (0.0)
No	14 (42.4)	12 (66.7)	2 (100.0)
***2***. ***How did you become aware of existing prediction rules that addressed the same clinical problem*?**			
Systematic review	18 (54.5)	9 (50.0)	2 (100.0)
No systematic review	7 (21.2)	8 (44.4)	0 (0.0)
No search	8 (24.2)	1 (5.6)	0 (0.0)
***3***. ***How important do you think it is to cite existing prediction rules for the same problem when deriving a new prediction rule*?**			
Important	30 (88.2)	17 (94.4)	2 (100.0)
Unimportant	4 (11.8)	1 (5.6)	0 (0.0)

CPR, clinical prediction rule.

^a^ the sum of responses for question 1 and 2 do not match the number of authors cited existing CPR because there was one author who did not respond for each question.

## Discussion

### Summary of main findings

The first objective of this study was to assess whether authors cited existing cardiovascular CPRs when developing new cardiovascular rules. We found many authors did not cite existing cardiovascular CPRs with only 56.5% of derivation studies citing at least one existing cardiovascular CPR. Our results also suggest that the citation of existing cardiovascular CPRs has improved over time.

Five categories of existing CPRs’ insufficiency was identified from the derivation studies. These included insufficiencies related to: (1) derivation, (2) construct, (3) performance, (4) transferability, and (5) evidence. The most commonly cited category (70.4%) was insufficiency related to the construct of existing CPRs such as issues in choice of predictor variables, outcomes, clinical usability, or presentation format. The next most common category (29.5%) was the insufficiency of existing CPRs’ transferability due to differences in population or setting of care.

Some authors justified the need for developing new cardiovascular CPRs by highlighting the insufficiencies of existing CPRs. Instead of creating new CPRs, some of these insufficiencies may be addressed by updating existing CPRs. For example, when an existing cardiovascular CPR is missing an important predictor variable, it may be updated by adding the predictor variable and recalculating the regression coefficients and intercept [17, 19, 20]. When an existing CPR does not perform well in a new population or setting, re-calibrating the intercept and slope or re-estimating coefficients may optimize the performance [19, 20]. When using a CPR is difficult because of complex calculations, presenting the CPR in a simpler and more user-friendly format (e.g. score chart) might resolve the problem [20, 68]. Lastly, some authors developed new CPRs mentioning transferability constraints of existing CPRs due to differences in key participant characteristics. The appropriate next step may be to conduct an external validation study to evaluate CPR performance, despite the differences in key participant characteristics [4, 69, 70]. The external validation study will also provide an opportunity to update the CPR in new populations [71]. If a new CPR was developed for every time there is a difference in characteristics, there will be numerous CPRs for the same problem.

We also evaluated why some authors cited existing CPRs in their derivation studies and others did not. However, the results of our survey did not explain the reasons for this difference: neither being aware of existing CPRs, having conducted a systematic review of existing CPRs, nor believing it is important to cite existing CPRs was clearly associated with citing existing cardiovascular CPRs.

One of the most interesting findings of the survey was that most authors believed citing existing CPRs was important with over 90% agreeing it was important to cite existing cardiovascular CPRs in derivation studies. Given this, it is difficult to justify the poor citation rate found in this study.

### Comparison with existing literature

The TRIPOD statement recommends authors present a rationale for developing a new CPR with references to existing CPRs [15]. We have shown that citation of existing CPRs is suboptimal in derivation studies of cardiovascular CPRs. This is similar to findings from previous reports that showed existing research evidence is often inadequately examined or ignored when conducting randomized controlled trials (RCTs) [72–75]. For example, Clarke and Hopewell [73] have shown that only 68.6% of RCTs published in four major general medicine journals discussed existing evidence in the introduction section by citing other clinical trials (28.6%) or a systematic review (40.0%).

It has been suggested that new research should be justified by discussing systematic reviews of existing evidence [13, 72, 73]. In our study, only three derivation studies (3.5%) cited a systematic review of existing cardiovascular CPRs [25, 38, 45] which is substantially less than citations of systematic reviews in RCTs [73]. Although more than half of authors who responded to our survey indicated they conducted a systematic review to identify existing CPRs, none of the included studies reported this. Ideally, authors of derivation studies should systematically review, discuss, and cite all existing CPRs relevant to clinical context as a part of justifying their research [46]. At the bare minimum, authors should refer to those CPRs of which they are aware or the best known in the field when conducting a systematic review is impractical.

### Strengths and limitations

To our knowledge, this is the first study to show that existing CPRs are inadequately cited in the introduction section of published derivation studies. We supplemented the review of derivation studies with a thematic content analysis and further augmented the understanding of problem by an author survey.

We used the International Register of Clinical Prediction Rules for Primary Care as the data source. Although published in 2014, the international register currently only includes CPRs published up to 2009. Therefore, it is important to consider that all derivation studies in our review predate the publication of TRIPOD statement and many of them predate the modern expectation that a systematic review of existing evidence should be done before any new research [13, 15, 72, 73]. In addition, we assessed only CPRs from the cardiovascular domain of the international register. Therefore, our findings may not be generalizable to the CPRs in other clinical areas or more recently developed CPRs.

The small number of the authors surveyed might have made it difficult to detect the associations between citation of existing CPR and the survey answers. We received responses from more than 70% of authors contacted by structuring a simple and short survey. The main tradeoff was that we could not include any open-ended questions that might have generated richer survey data and led to more in-depth analysis of the issue. Recall bias should also be considered when interpreting our survey results because two questions relied on authors’ recollection of information from past CPR development.

Some authors indicated in their survey response that they were unaware of an existing CPR at the time of derivation which was at odds with the review of their derivation studies which demonstrated that existing CPRs were cited. These discrepancies might have arisen from the different interpretations of the term “same clinical problem” in the first survey question. Some of these authors acknowledged there were existing CPRs for the problems in introduction sections but indicated existing CPRs were inapplicable to their populations or practice settings. The differences in setting or population that existing CPRs were developed might have lead some authors to determine these CPRs did not address the “same clinical problem”.

### Research and clinical implications

We speculate that researchers keep deriving new CPRs when CPRs already exist for the same clinical problem for the following reasons. Firstly, researchers may be more familiar with methods for developing a new CPR (e.g. logistic regression) than methods needed to update an existing CPR (e.g. recalibration). Secondly, clinical data for developing a CPR are increasingly available from widespread use of electronic health records [76]. Thirdly, researchers may be attracted to creating a new CPR because they view this as more novel and therefore more likely to be published in higher impact journals. Lastly, there were no standardized reporting guidelines for authors to provide a clear rationale for developing a new CPR before the publication of the TRIPOD statement in 2015.

CPRs are notably more common for some conditions (e.g. cardiovascular, respiratory, musculoskeletal) but seldom developed for many clinical areas (e.g. eye, ear, pregnancy related) [7]. Researchers should systematically search for any existing CPRs relevant to their context before deciding to develop a new CPR. When there is no existing CPR for the problem and there is an unmet clinical need, developing a CPR is well justified.

When a systematic evaluation of existing CPRs identifies a well-built CPR with good performance in external validations, researchers should focus on implementing the CPR and assessing the impact in clinical practice. Then clinicians can confidently adopt a CPR that has been externally validated and proven to improve relevant clinical outcomes. For example, the Ottawa ankle rules showed robust predictive performance in wide range of populations and settings [77–79] and reduced utilization of radiography without jeopardizing clinical outcomes in impact studies [80–83]. Many GPs recognize and use Ottawa ankle rules in clinical practice despite lack of clinical guidelines on use of these CPRs at the point of care [9]. When updated, the International Register of CPRs can be an important source for setting research priorities and identifying CPRs ready to be tested for implementation in clinical practice.

When existing CPRs for the clinical problem are found but some insufficiencies are also identified, researchers should attempt to resolve these insufficiencies by validating and updating the existing CPR. When it is not possible to resolve these insufficiencies, developing a new CPR can be justified. This approach may reduce research waste in CPR development in the following ways. Firstly, the knowledge generated from researchers’ time and efforts in derivation studies is used in planning new research. When the only reason participants were recruited was to develop a new CPR, their contribution is not thrown away. Lastly, this approach may prevent unnecessarily adding new CPRs for the same clinical problem.

## Conclusion

This is the first study to demonstrate that existing CPRs are poorly cited in the derivation studies of cardiovascular CPRs. Almost all authors of cardiovascular CPR believe citing existing CPRs is important and the TRIPOD statement also recommends to do so. Authors developing a new cardiovascular CPR should provide a clear rationale with citations to existing CPRs to avoid unnecessary duplication.

## Supporting information

S1 Appendix Questionnaire for authors of cardiovascular prediction rules. (TIF)

S1 Data Insufficiencies of existing cardiovascular prediction rules. (XLSX)

S1 Table Characteristics of derivation studies that authors responded, did not respond and could not be contacted. (DOCX)
