# Supplementary figures and images for: Why do authors derive new cardiovascular clinical prediction rules in the presence of existing rules? A mixed methods study
Source: PLoS One. 2017 Jun 7;12(6):e0179102. doi: 10.1371/journal.pone.0179102 (PMC5462434; doi:10.1371/journal.pone.0179102)

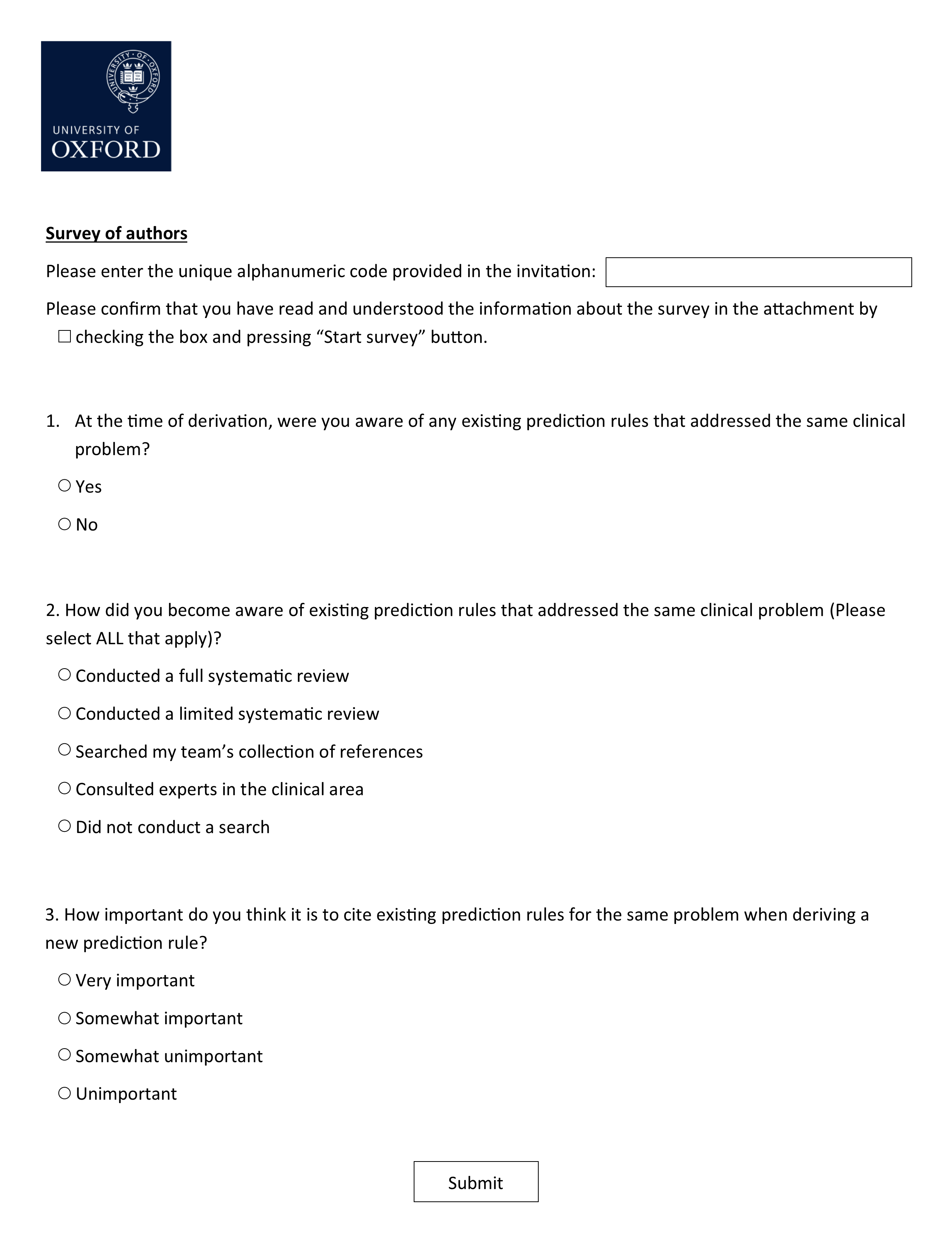

Supplement: S1 Appendix — (TIF) [file pone.0179102.s001.tif]
