# Supplementary material for: Why do authors derive new cardiovascular clinical prediction rules in the presence of existing rules? A mixed methods study
Source: PLoS One. 2017 Jun 7;12(6):e0179102. doi: 10.1371/journal.pone.0179102 (PMC5462434; doi:10.1371/journal.pone.0179102)
Supplement: S1 Table — (DOCX) [file pone.0179102.s003.docx]

**S1 Table. Characteristics of derivation studies that authors responded, did not respond and could not be contacted.**

| Characteristics | Author contacted by e-mail or post | | No author could be contacted | Total |
| --- | --- | --- | --- | --- |
|  | **Response received** | **Response not received** |  |  |
| Number of derivation study | 54 | 22 | 9 | 85 |
| Derivation year, median (IQR) | 2002 (1997 – 2005) | 1998 (1989 – 2005) | 2001 (1998 – 2005) | 2001 (1995 – 2005) |
| Type of CPR, n (%) |  |  |  |  |
| Diagnostic | 24 (44.4) | 15 (68.2) | 3 (33.3) | 42 (49.4) |
| Prognostic | 30 (55.6) | 7 (31.8) | 6 (66.7) | 43 (50.6) |
| Sub-domain, n (%) |  |  |  |  |
| K90 Stroke/cerebrovascular accident | 11 (20.4) | 1 (4.5) | 2 (22.2) | 14 (16.5) |
| K22 Risk factor cardiovascular disease | 8 (14.8) | 2 (9.1) | 1 (11.1) | 11 (12.9) |
| K77 Heart failure | 5 (9.3) | 4 (18.2) | 0 (0.0) | 9 (10.6) |
| K93 Pulmonary embolism | 6 (11.1) | 2 (9.1) | 1 (11.1) | 9 (10.6) |
| K74 Ischemic heart disease with angina | 4 (7.4) | 2 (9.1) | 1 (11.1) | 7 (8.2) |
| K75 Acute myocardial infarction | 1 (1.9) | 5 (22.7) | 1 (11.1) | 7 (8.2) |
| K94 Phlebitis/thrombophlebitis | 5 (9.3) | 1 (4.5) | 0 (0.0) | 6 (7.1) |
| Other | 14 (25.9) | 5 (22.7) | 3 (33.3) | 22 (25.9) |
| Location, n (%) |  |  |  |  |
| USA | 22 (40.7) | 14 (63.6) | 2 (22.2) | 38 (44.7) |
| UK | 8 (14.8) | 3 (13.6) | 1 (11.1) | 12 (14.1) |
| Europe | 7 (13.0) | 1 (4.5) | 4 (44.4) | 12 (14.1) |
| Canada | 5 (9.3) | 0 (0.0) | 0 (0.0) | 5 (5.9) |
| Multiple | 10 (18.5) | 1 (4.5) | 1 (11.1) | 12 (14.1) |
| Other | 2 (3.7) | 3 (13.6) | 1 (11.1) | 6 (7.1) |
| Citation of existing CPR, n (%) |  |  |  |  |
| Cited existing CPR | 34 (63.0) | 9 (40.9) | 5 (55.6) | 48 (56.5) |
| Did not cite existing CPR | 18 (33.3) | 12 (54.5) | 3 (33.3) | 33 (38.8) |
| No existing CPR to cite | 2 (3.7) | 1 (4.5) | 1 (11.1) | 4 (4.7) |

CPR, clinical prediction rule.
